# Massive Pilomatrixoma of the Scalp: A Case Report

**DOI:** 10.7759/cureus.54511

**Published:** 2024-02-20

**Authors:** Rodrigo Duarte, Bruna Pimentel, Filipa D Miranda, Manuel Gonçalves, João Pereira

**Affiliations:** 1 Internal Medicine, Centro Hospitalar Lisboa Ocidental, Lisbon, PRT; 2 Diabetes and Endocrinology, Centro Hospitalar Lisboa Ocidental, Lisbon, PRT

**Keywords:** malherbe's calcifying epithelioma, scalp tumor, clinical case report, calcified epithelioma of malherbe, pilomatrixoma

## Abstract

Pilomatrixoma, also called epithelioma of Malherbe, is a benign neoplasm derived from hair follicle matrix cells. It usually presents as a solitary mass in the head and neck region and is more frequent in children and young adults, females, and the Caucasian population. Lesions equal to or greater than 5 cm are categorized as giant pilomatrixomas. We present a case of a 75-year-old female, with no known medical history, who was brought to the emergency department (ED) after falling on the street. She had a giant soft head tissue tumor, severe anemia due to intralesional chronic small hemorrhages and folates and cobalamin deficiencies, and delirant speech. The anatomopathological result of the biopsy of the tumor revealed to be a pilomatrixoma. The patient was then referred to plastic surgery, with complete excision of the tumor. After surgery, she was transferred to the psychiatric team, who assumed the delirant speech to be in the context of schizophrenia. She was discharged four months after admission.

## Introduction

Pilomatrixoma, also known as calcifying epithelioma of Malherbe, is a benign subcutaneous tumor that originated from the hair follicle matrix, which mostly occurs on the head and neck [1-8]. It exhibits a bimodal distribution, affecting individuals under 30 years and in their sixth and seventh decade of life [1-3]. It is more frequent in female individuals and the Caucasian population [1,8]. The incidence is not precise, but it is reported between one and three in 1,000 dermatohistopathologic specimens examined [1]. It usually presents as a solitary firm painless subcutaneous slow-growing mass, attached to the skin with no invasion of the underlying tissue [1,2,5,8]. Its dimensions often range from 0.5 to 3 cm, but lesions greater than 5 cm in diameter can be seen and are described as giant pilomatrixoma [3,5]. Those can appear reddish/bluish, ulcerated, and even with a necrotic area [5]. Pilomatrixomas are not known for spontaneous regression; thus, complete surgical excision is the treatment of choice and often curative [2,3,5]. We present a unique case of a female patient with a massive pilomatrixoma of the scalp, severe anemia, and schizophrenia. The patient has been informed and has consented to the documentation of this case report.

## Case presentation

**Figure 1:**
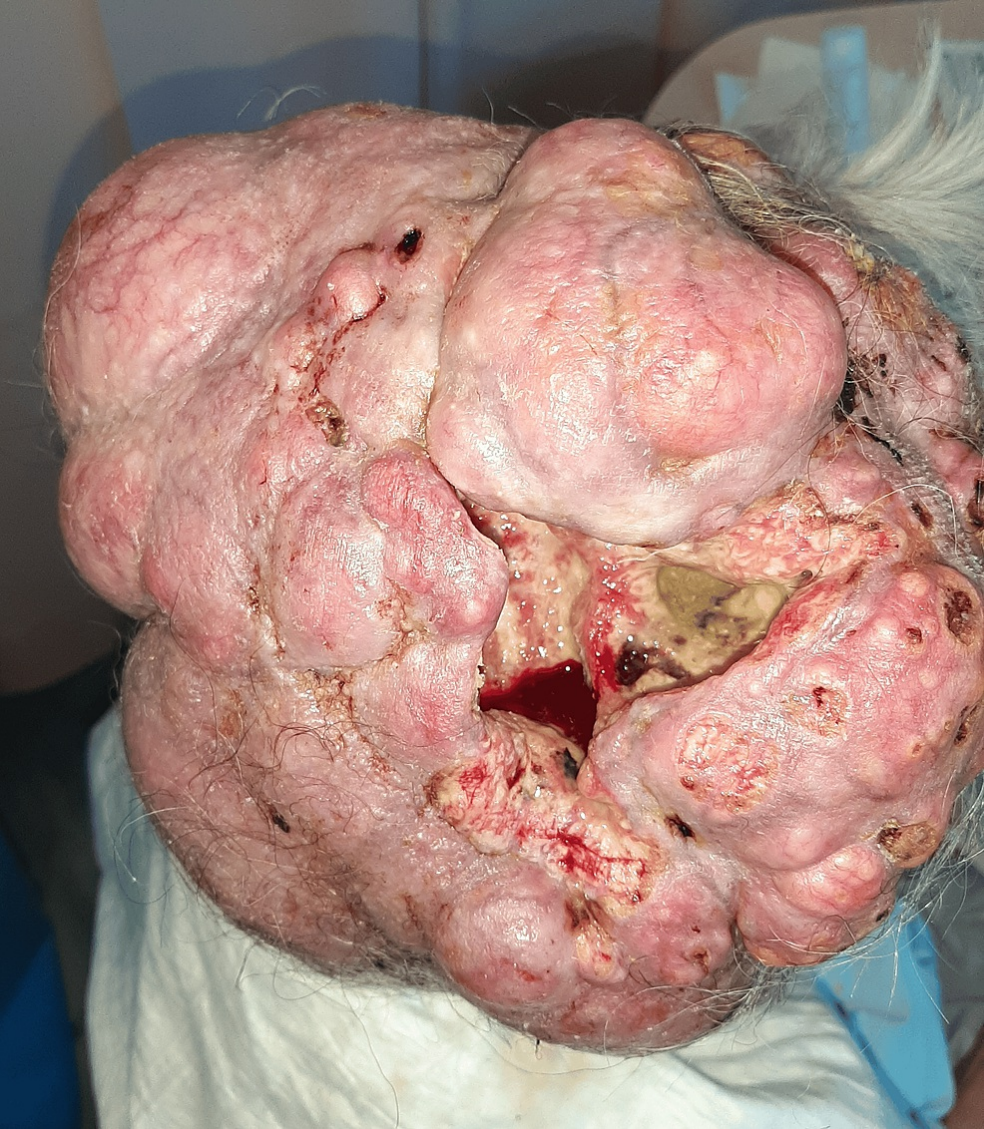
Giant scalp mass with a necrotic center, where a hemorrhage can be visualized. Multiple areas of superficial friability and erosion can be observed.

**Figure 2:**
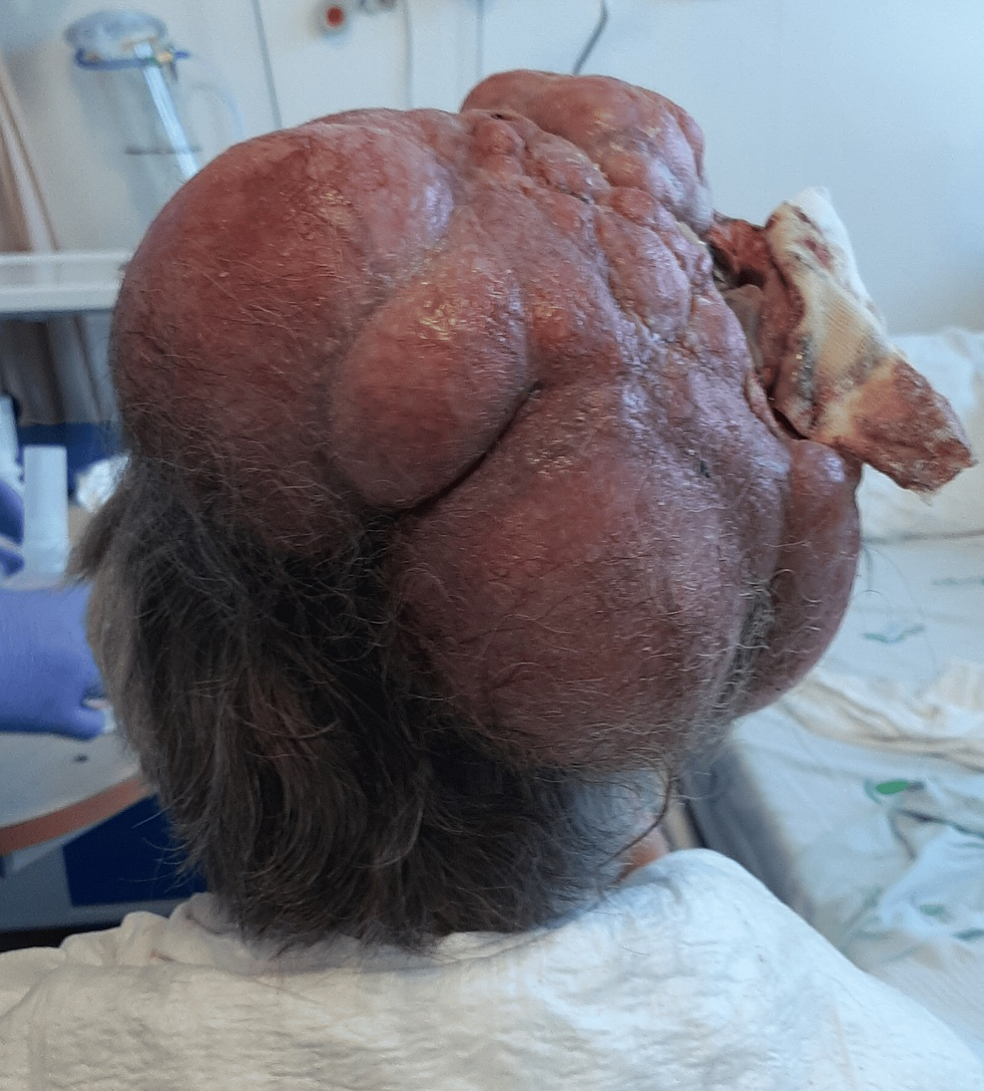
Posterior view of the patient's head, where a large predominantly right-sided mass can be seen.

A 75-year-old female, with no known medical records, presents to the emergency department (ED) after fainting, with loss of consciousness, on the street. She had a massive exophytic lesion on her right parieto-occipital region. A fragile social context was notorious with important nutritional deficiencies, as she was dehydrated, with an intense smell, admitting to consuming only cookies for sustenance. During the examination, the patient demonstrated orientation in person, time, and space. She presented delusional speech characterized by a rapid flow of ideas (flight of ideas) and held an erotomaniac and persecutory delusion, referring that "the neighbors wanted to rob me, leaving erotic messages on the walls." Her vitals were as follows: blood pressure, 124/66 mmHg; pulse, 56 bpm; oxygen saturation, 96% at room air; and afebrile. There was a right parieto-occipital exophytic lesion of huge dimensions, with a painless erythematous rash, soft to palpation, with fluctuation and multilobulated. The lesion had a central ulcer with a purulent exudate and a fetid and intense smell (Figures 1, 2). Due to her delusional speech, it was not possible to determine for how long the lesion was present.

On blood analysis, microcytic anemia (hemoglobin: 2.8 g/dL, mean globular volume: 49.8 fL) was evident, with iron and folate deficiency (Table 1). No urinary toxins were present.

**Table 1:** Laboratory results

Laboratory results	Results	Reference values
Hemoglobin (g/dL)	2.8	12-15
Hematocrit (%)	11.1	36-46
Mean glomerular volume (fL)	49.8 fL	80-96.1
Leucocytes (cells/uL)	11,100	4,000-10,000
Neutrophils (%)	80.7	40-80
Lymphocytes (%)	11.8	20-40
Monocytes (%)	6.6	2-11.7
Eosinophils (%)	0.2	1-6
Basophils (%)	0.7	0-2
Platelets (cells/uL)	630,000	150,000-400,000
Iron (mcg/dL)	14	33-193
Ferritin (ng/mL)	17.4	30-340
Total iron-binding capacity (mcg/dL)	381	250-425
Transferrin saturation (%)	4	20-45
Folic acid (nmol/L)	6.23	10-42
Cobalamin (pmol/L)	157	141-489
Glucose (mg/dL)	143	74-106
Urea (mg/dL)	36	17-49
Creatinine (mg/dL)	1.29	0.50-0.90
Sodium (mmol/L)	139	136-145
Potassium (mmol/)	4.38	3.50-5.10
Chlorine (mmol)	105	98-105
C-reactive protein (mg/dL)	0.38	<0.50
Procalcitonin (ng/mL)	0.48	<0.1

**Figure 3:**
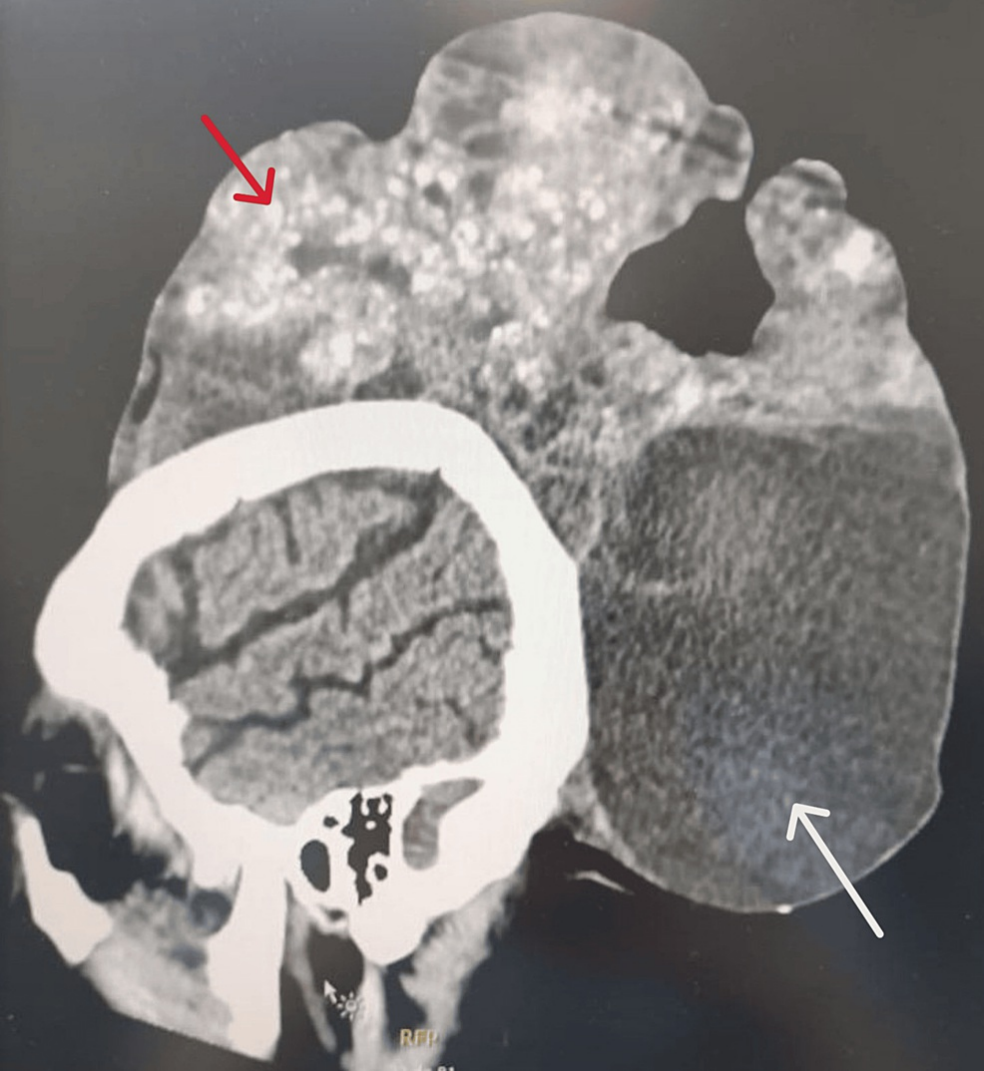
Lateral view of the patient's head on the CT scan, which showed a lesion of 21 cm in transverse axis, 16.5 cm in longitudinal axis, and 16 cm in anteroposterior axis. A cystic formation (white arrow) can be noted, as well as some calcified areas on the lesion (red arrow). CT: computed tomography

**Figure 4:**
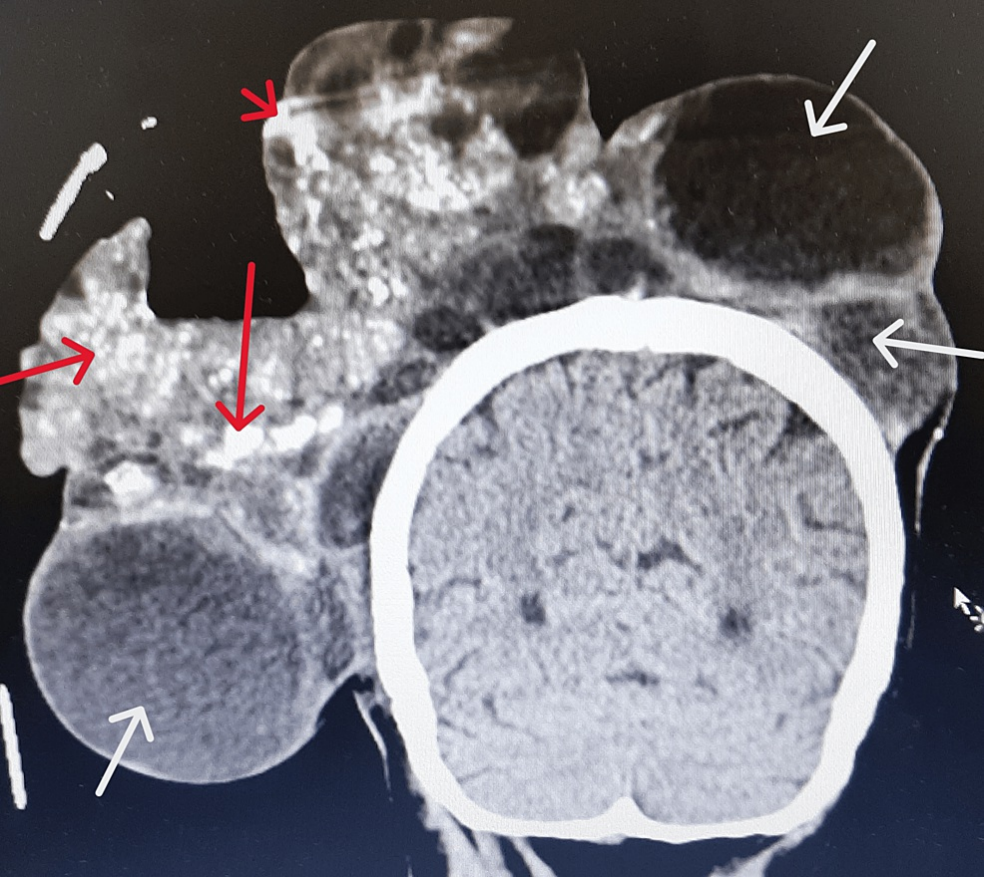
Anteroposterior view of the lesion on CT scan. Some cystic formations (white arrows) can be noted, as well as some calcified hyperdense areas on the lesion (red arrows). CT: computed tomography

**Figure 5:**
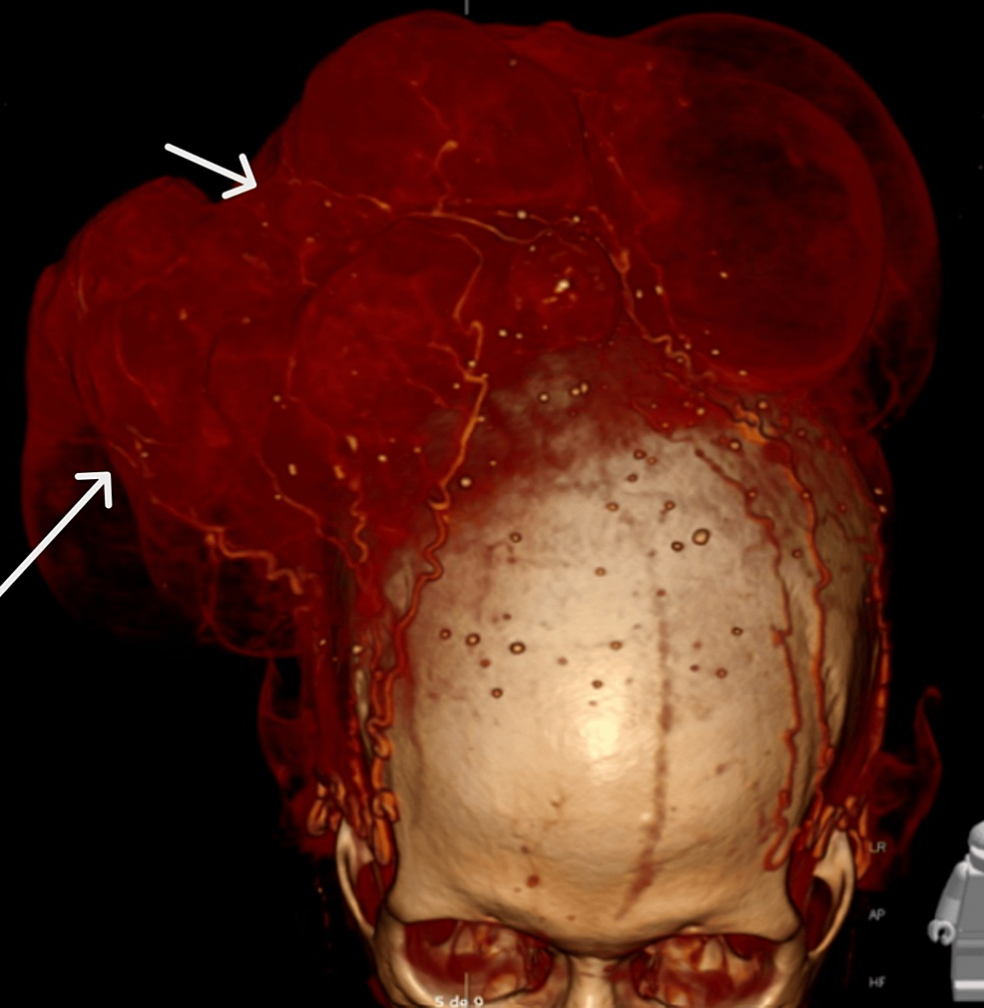
CT scan reconstitution of the tumor and its dense vascular supply (white arrows). CT: computed tomography

On computed tomography (CT), a right parieto-occipital massive expansive lesion of the scalp was shown, multiloculated/multicystic with partially calcified hyperdense areas and with a potential central necrotic hub. It measured 21 cm in transverse axis, 16.5 cm in longitudinal axis, and 16 cm in anteroposterior axis. No bone erosion or intracranial involvement was shown, and it had an incipient pattern of microvascular chronic ischemic leukoencephalopathy (Figures 3, 4). On CT angiography scan, a dense extracranial vascular network was visible (Figure 5).

Three packs of red blood cells were administered in the emergency department, resulting in an increase in hemoglobin level to 7.3 g/dL, prior to being transferred to the medical ward. The anemia was classified secondary to the chronic blood loss and to iron and folate deficiency, and the patient was given 1 g of ferric carboxymaltose and started on folate 5 mg per day. Cranial magnetic resonance imaging and full body computed tomography were done, with no evidence of metastatic lesions. The biopsy of the scalp lesion, done by plastic surgery, showed an intradermal tumor known as pilomatrixoma, with no malignancy or histopathologic signs associated.

In the medical ward, she was observed by the psychiatric team who assumed the delusional speech was due to schizophrenia. Following clinical stabilization and an increase in hemoglobin levels to 10.9 g/dL due to supplementation with hematinic factors, the patient underwent elective excision of the lesion and application of a skin coating. During the surgery, there was a period of severe hypotension (31/15 mmHg) and tachycardia (110 bpm) in the context of blood loss volume superior to the predicted that was reverted after the transfusion of two red blood cell packs. After the procedure, the recovery was uneventful. The patient was then transferred to the psychiatric ward and started on paliperidone, with a decreased frequency of her psychiatric symptoms. She was discharged four months after the initial hospital admission and medicated with paliperidone for the schizophrenia.

## Discussion

Giant pilomatrixoma is a rare distinctive clinical variant of pilomatrixoma [6]. Its size is variable, being 34 cm in diameter the largest reported lesion to date [3]. According to Sabater-Abad et al., nearly 40% of giant pilomatrixomas are ulcerated. Due to these characteristics, the differential diagnosis is challenging, including malignant neoplasms such as cutaneous squamous cell carcinoma, dermatofibrosarcoma protuberans, and cutaneous metastases [6].

The singularity of the reported case resides not only in its dimensions but also in its complexity and the hemodynamic impact associated. The diagnosed psychopathology had important implications for the progression of the disease, as it altered the perception of the tumor, with the eviction of medical care, allowing its growth during an unspecified period of time. Additionally, we suspect that it had an impact on the patient's diet, potentially contributing to the observed deficiencies in hematinic factors.

Furthermore, as indicated in the CT scan, the presence of a dense tumoral vascular network and a necrotic hub, along with intratumoral hemorrhage, was considered a significant contributor to the patient's anemia. This anemia was determined to be severe and chronic, resulting from iron deficiency caused by chronic small intralesional hemorrhages and a lack of folates and cobalamin, as inadequate nutrient intake also played a crucial role in the severity of the anemia. However, after administering iron and folate replacements, the patient experienced a recovery in hemoglobin levels, eliminating the need for further blood transfusions. Additionally, the vascular network posed a substantial risk for surgical hemorrhage, which was effectively managed through the administration of red blood cell packs and fluid restoration.

## Conclusions

The present case shows a rare benign tumor of the hair follicle matrix cells, unique by its giant dimensions and the hemodynamic repercussions associated. The psychopathology of the patient, with a long period of eviction from medical care, allowed the tumors' massive growth, developing a dense vascular supply, a source of chronic blood loss, and the syncope that brought her to the ER. Therefore, the exceptional nature of the case lay in the remarkable size observed, its multitude of pathologies, and its intricate complexity, necessitating the coordinated efforts of a multidisciplinary team to optimize its resolution and improve the patient's overall outcome.
